# Sequence of Epinephrine and Advanced Airway Placement After Out-of-Hospital Cardiac Arrest

**DOI:** 10.1001/jamanetworkopen.2023.56863

**Published:** 2024-02-19

**Authors:** Masashi Okubo, Sho Komukai, Junichi Izawa, Kosuke Kiyohara, Tasuku Matsuyama, Taku Iwami, Tetsuhisa Kitamura

**Affiliations:** 1 Department of Emergency Medicine, University of Pittsburgh School of Medicine, Pittsburgh, Pennsylvania; 2 Division of Biomedical Statistics, Department of Integrated Medicine, Graduate School of Medicine, Osaka University, Osaka, Japan; 3 Department of Internal Medicine, Okinawa Prefectural Chubu Hospital, Okinawa, Japan; 4 Department of Food Science, Otsuma Women’s University, Tokyo, Japan; 5 Department of Emergency Medicine, Kyoto Prefectural University of Medicine, Kyoto, Japan; 6 Department of Preventive Services, Kyoto University, Kyoto, Japan; 7 Division of Environmental Medicine and Population Sciences, Department of Social and Environmental Medicine, Osaka University Graduate School of Medicine, Osaka, Japan

## Abstract

**Importance:**

While epinephrine and advanced airway management (AAM) (supraglottic airway insertion and endotracheal intubation) are commonly used for out-of-hospital cardiac arrest (OHCA), the optimal sequence of these interventions remains unclear.

**Objective:**

To evaluate the association of the sequence of epinephrine administration and AAM with patient outcomes after OHCA.

**Design, Setting, and Participants:**

This cohort study analyzed the nationwide, population-based OHCA registry in Japan and included adults (aged ≥18 years) with OHCA for whom emergency medical services personnel administered epinephrine and/or placed an advanced airway between January 1, 2014, and December 31, 2019. The data analysis was performed between October 1, 2022, and May 12, 2023.

**Exposure:**

The sequence of intravenous epinephrine administration and AAM.

**Main Outcomes and Measures:**

The primary outcome was 1-month survival. Secondary outcomes were 1-month survival with favorable functional status and prehospital return of spontaneous circulation. To control imbalances in measured patient demographics, cardiac arrest characteristics, and bystander and prehospital interventions, propensity scores and inverse probability of treatment weighting (IPTW) were performed for shockable and nonshockable initial rhythm subcohorts.

**Results:**

Of 259 237 eligible patients (median [IQR] age, 79 [69-86] years), 152 289 (58.7%) were male. A total of 21 592 patients (8.3%) had an initial shockable rhythm, and 237 645 (91.7%) had an initial nonshockable rhythm. Using IPTW, all covariates between the epinephrine-first and AAM-first groups were well balanced, with all standardized mean differences less than 0.100. After IPTW, the epinephrine-first group had a higher likelihood of 1-month survival for both shockable (odds ratio [OR], 1.19; 95% CI, 1.09-1.30) and nonshockable (OR, 1.28; 95% CI, 1.19-1.37) rhythms compared with the AAM-first group. For the secondary outcomes, the epinephrine-first group experienced an increased likelihood of favorable functional status and prehospital return of spontaneous circulation for both shockable and nonshockable rhythms compared with the AAM-first group.

**Conclusions and Relevance:**

These findings suggest that for patients with OHCA, administration of epinephrine before placement of an advanced airway may be the optimal treatment sequence for improved patient outcomes.

## Key Points

**Question:**

What sequence of intravenous epinephrine administration and advanced airway placement is associated with improved patient outcomes after out-of-hospital cardiac arrest (OHCA)?

**Findings:**

In this cohort study of 259 237 Japanese adult patients with OHCA for whom emergency medical services personnel administered intravenous epinephrine and/or placed an advanced airway, epinephrine as a first strategy was associated with a higher likelihood of 1-month survival in both shockable and nonshockable rhythms than advanced airway management as the first strategy.

**Meaning:**

These findings suggest that for patients with OHCA, administration of epinephrine before placement of an advanced airway may be the optimal treatment sequence for improved patient outcomes.

## Teaser

This cohort study evaluates the association of the sequence of epinephrine administration and advanced airway placement with patient outcomes after out-of-hospital cardiac arrest in Japan.

## Introduction

Out-of-hospital cardiac arrest (OHCA) is an important public health problem, with high mortality rates observed globally.^1^ Emergency medical services (EMS) play a pivotal role in providing initial treatment for patients with OHCA as a part of the chain of survival.^2^ Prehospital care entails a multifaceted approach, encompassing simultaneous and sequential resuscitative interventions, such as chest compressions, airway management, ventilation, defibrillation, and medication administration.^2^

As a part of prehospital care, advanced life support interventions, including epinephrine administration and advanced airway placement (ie, supraglottic airway [SGA] insertion and endotracheal intubation) are commonly performed.^2,3^ The 2020 American Heart Association guidelines for cardiopulmonary resuscitation and emergency cardiovascular care suggest that “it may be reasonable to administer epinephrine after initial defibrillation attempts have failed for a shockable rhythm”^2^^(pS396)^ (weak recommendation, limited data), and “for a nonshockable rhythm, it is reasonable to administer epinephrine as soon as feasible”^2^^(pS396)^ (moderate recommendation, limited data). The American Heart Association resuscitation guidelines also note that the timing of advanced airway management (AAM) requires consideration of a host of patient and health care professional characteristics.^2^ Ultimately, the optimal sequence for epinephrine administration and AAM remains insufficiently examined. The International Liaison Committee on Resuscitation^4^ consensus on cardiopulmonary resuscitation and emergency cardiovascular care science with treatment recommendations identified the optimal time point during cardiopulmonary resuscitation (CPR) to change to different airway management techniques (eg, from bag-mask ventilation to AAM) as one of the current knowledge gaps. Consequently, our objective was to evaluate the association of the sequence of intra-arrest epinephrine administration and AAM with patient outcomes after OHCA, comparing the epinephrine-first strategy with the AAM-first strategy.

## Methods

### Study Design and Setting

In this cohort study, we conducted a retrospective analysis of the All-Japan Utstein Registry, a nationwide, population-based OHCA registry.^5,6^ The registry prospectively collects EMS-assessed OHCAs using the Utstein Resuscitation Registry Template for OHCA.^7,8,9^ The details of the registry have been previously reported.^10,11,12^ The institutional review board of the Osaka University Graduate School of Medicine approved this study and waived the need for informed consent because of deidentified data. We followed the Strengthening the Reporting of Observational Studies in Epidemiology (STROBE) reporting guideline.

Cardiac arrest was defined as the absence of cardiac mechanical activity, confirmed by a lack of clinical evidence of circulation.^8,9^ The collected variables included age, sex, date and time of cardiac arrest, etiologies of cardiac arrest, onset witnessed by a bystander, first documented rhythms, presence and type of bystander CPR (chest compression only without rescue breathing or conventional CPR with rescue breathing), dispatcher CPR instruction, public-access automated external defibrillator shock delivery, type of AAM, prehospital administration of intravenous (IV) fluids and epinephrine, and resuscitation time course, as well as outcome measures, including prehospital return of spontaneous circulation (ROSC), 1-month survival, and functional status at 1 month after the cardiac arrest. The resuscitation time course variables included each receipt of an emergency call, initiation of CPR by EMS personnel, defibrillation by EMS personnel, epinephrine administration, prehospital ROSC, successful placement of an advanced airway device, and hospital arrival. These time variables were recorded in minutes according to the time on the clock used by each EMS system.

In Japan, municipal governments organize EMS systems through local fire departments, and each ambulance crew has 3 EMS personnel, including at least 1 emergency life-saving technician (ELST) who completed extensive training in prehospital care. The EMS personnel follow the Japanese resuscitation guidelines, which are based on the International Liaison Committee on Resuscitation consensus statements.^13,14^ All ELSTs are authorized to insert an IV line, administer IV epinephrine, and place an SGA (eg, laryngeal tube and laryngeal mask) for patients with OHCA under online medical direction by a consulting physician.^5,6,10^ The ELSTs are not permitted to obtain an intraosseous (IO) route access. Although all ELSTs can place an SGA, only certified ELSTs are permitted to perform endotracheal intubation (ETI) for patients with OHCA under online medical direction after additional training.^5,6,10^ To become the certified ELST capable of performing ETI, each ELST must complete a training program authorized by their regional medical control committee.^5,6,10^ The training period includes more than 62 terms, and each term consists of a 50-minute training session.^15,16^ The practical training includes more than 30 successful intubations in operating rooms under the guidance and supervision of attending physicians.^15,16^ The sequence of epinephrine administration and AAM was at the discretion of EMS personnel under online medical direction.

### Study Participants

We examined data of all patients with OHCA in the All-Japan Utstein Registry from January 1, 2014, through December 31, 2019, representing the most recent data available at the time of analysis. The inclusion criteria for this study were as follows: age of 18 years or older, cardiac arrest occurring before EMS arrival, attempted resuscitation by EMS personnel, and cardiac arrest attended by an ELST. Attempted resuscitation was defined as external shock delivery by EMS personnel or a bystander and/or chest compressions by EMS personnel.^7,8,9^

We excluded patients who (1) had unknown epinephrine administration status, (2) had unknown AAM status, (3) did not receive epinephrine or AAM, (4) had unknown timing of epinephrine administration or AAM, (5) had an unknown initial rhythm, (6) had unknown covariates (described below), (7) received epinephrine or AAM after first ROSC, or (8) had an interval from emergency call to initiation of EMS CPR of longer than 30 minutes.^10^ The eligible patients, therefore, received epinephrine and/or AAM. We did not define eligibility as patients who received epinephrine and AAM because if either intervention was associated with outcomes and a patient had ROSC after 1 intervention without the other intervention, the patient should not be excluded from the analysis; otherwise, selection bias would be introduced.

Because the timing of epinephrine administration and AAM were recorded in minutes, we were not able to determine the sequence of interventions if a patient received epinephrine and AAM within the same whole minute. In the primary analysis, we further excluded patients who received epinephrine and AAM in the same whole minute. In the 2 sensitivity analyses, patients who received epinephrine and AAM within the same whole minute were included in either the AAM-first group or epinephrine-first group.

### Exposure and Outcomes

The main exposure was the sequence of IV epinephrine administration and AAM. The primary outcome was 1-month survival. Secondary outcomes included prehospital ROSC and 1-month survival with favorable functional status, defined as a Cerebral Performance Category (CPC) scale score of 1 or 2,^7,8,9^ as assessed by a physician responsible for each patient’s care 1 month after successful resuscitation. A CPC score of 1 represents good cerebral performance; CPC 2, moderate cerebral disability; CPC 3, severe cerebral disability; CPC 4, coma or vegetative state; and CPC 5, death or brain death.^7^ To collect data on survival and functional outcomes, EMS personnel in charge followed up with all patients and had interviews with physicians 1 month after the cardiac arrest.^10,12^

### Statistical Analyses

Analyses were conducted between October 1, 2022, and May 12, 2023. We stratified the patients into 2 subcohorts based on their initial rhythm on EMS arrival: shockable (ventricular defibrillation or pulseless ventricular tachycardia) or nonshockable (pulseless electrical activity or asystole) rhythm, because current resuscitation guidelines recommend 2 algorithms according to the initial rhythm.^2^ Continuous variables are presented as medians with IQRs, and categorical variables are presented as counts with proportions. We report standardized mean differences to describe differences in baseline patient characteristics.

To account for the differences in characteristics between patients who were administered epinephrine first and who received AAM first, we calculated propensity scores and performed inverse probability of treatment weighting (IPTW) in each subcohort of the initial rhythms.^17,18,19^ A propensity score was generated for each patient using a logistic regression model. The dependent variable was epinephrine first (vs AAM first). In the propensity score model, as covariates, we included age, sex, year, season, day (weekday or weekend), time (daytime, 9:00 am to 4:59 pm; nighttime, 5:00 pm to 8:59 am), etiology (medical or nonmedical), witness status (witnessed or unwitnessed), initial rhythm (pulseless electrical activity or asystole only in the nonshockable subcohort), bystander CPR (chest compression only, chest compression with assist ventilation, or none), public access automated external defibrillator shock delivery (only in the shockable subcohort), dispatcher CPR instruction, prehospital physician involvement, the interval between the emergency call and initiation of CPR by EMS personnel, and the interval between initiation of CPR by EMS personnel and first treatment (epinephrine administration or AAM). The etiology of arrest was presumed to be medical unless the etiology was trauma, drug overdose, drowning, electrocution, or asphyxia using the definitions in the Utstein Resuscitation Registry Template.^7^ The interval between initiation of CPR by EMS personnel and first treatment was included to account for the potential different distributions of time to the first treatment between patients who were administered epinephrine first and who received AAM first and to address resuscitation time bias.^20^ If the time to the first treatment is not accounted for, the group with a longer time to the first treatment would have a longer time for which patients cannot achieve ROSC and would be biased toward harmful association (resuscitation time bias).^20^ These covariates were chosen based on the known association with the outcomes, biological plausibility, and adequate ascertainment.^2,21,22,23^

In each subcohort of the initial rhythm, using the calculated propensity scores, we performed IPTW to control imbalances in measured covariates between the epinephrine-first group and AAM-first group using a sandwich variance estimator to estimate the variance for the calculated IPTW estimates.^24^ We assessed the magnitude of covariate imbalances using standardized mean differences and regarded a standardized mean difference less than 0.100 as a well-matched balance.^18^ Using the weighted population, we fit logistic regression models to evaluate the associations of the sequence of epinephrine administration and AAM with outcomes.

We conducted 2 sensitivity analyses. First, patients who received epinephrine and AAM within the same whole minute were included in the AAM-first group (sensitivity analysis 1). Second, patients who received epinephrine and AAM within the same whole minute were included in the epinephrine-first group (sensitivity analysis 2). All statistical analyses were performed using R, version 4.1.1 software (R Foundation for Statistical Computing).

## Results

We observed 744 566 adult patients with OHCA in Japan from 2014 through 2019 (Figure). After applying the inclusion and exclusion criteria, 259 237 patients were eligible for our study. Of the eligible patients, the median (IQR) age was 79 (69-86) years, and 152 289 (58.7%) were male and 106 948 (41.3%) female. A total of 21 592 patients (8.3%) presented with an initial shockable rhythm, and 237 645 (91.7%) presented with an initial nonshockable rhythm. Baseline patient characteristics in each subcohort of initial rhythm are presented in Table 1. Among the 21 592 patients with an initial shockable rhythm, 6221 (28.8%) received epinephrine first, 14 980 (69.4%) received AAM first, and 391 (1.8%) received epinephrine and AAM in the same whole minute. Among the 237 645 patients with an initial nonshockable rhythm, 41 435 (17.4%) received epinephrine first, 193 372 (81.4%) received AAM first, and 2838 (1.2%) received epinephrine and AAM in the same whole minute. In the shockable rhythm subcohort, the median (IQR) time from the initiation of CPR by EMS personnel to epinephrine administration was 11 (8-15) minutes in the epinephrine-first group and 14 (10-19) minutes in the AAM-first group, while the median (IQR) time from the initiation of CPR by EMS personnel to AAM was 14 (10-19) minutes in the epinephrine-first group and 7 (5-11) minutes in the AAM-first group. In the nonshockable rhythm subcohort, the median (IQR) time to epinephrine was 12 (9-16) minutes in the epinephrine-first group and 15 (10-20) minutes in the AAM-first group, while the median (IQR) time to AAM was 14 (10-19) minutes in the epinephrine-first group and 8 (5-11) minutes in the AAM-first group. In both initial rhythm subcohorts, the majority of patients received an SGA for AAM.

**Figure.**
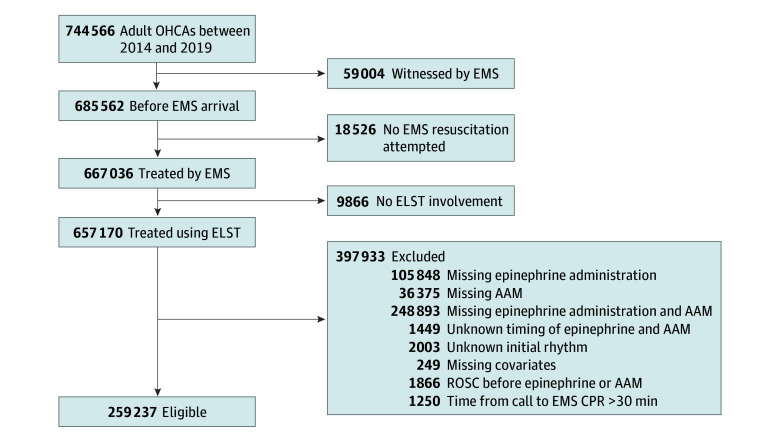
Study Flowchart AAM indicates advanced airway management; CPR, cardiopulmonary resuscitation; ELST, emergency life-saving technician; EMS, emergency medical services; OHCA, out-of-hospital cardiac arrest; ROSC, return of spontaneous circulation.

**Table 1.** Characteristics of Adult Patients With Out-of-Hospital Cardiac Arrest Stratified by Timing of Epinephrine and Advanced Airway Management

	Shockable rhythm, No. (%) of patients (n = 21 592)			Standardized mean difference^a^	Nonshockable rhythm, No. (%) of patients (n = 237 645)			Standardized mean difference^a^
	Epinephrine first (n = 6221)	AAM first (n = 14 980)	Epinephrine and AAM in the same whole minute (n = 391)		Epinephrine first (n = 41 435)	AAM first (n = 193 372)	Epinephrine and AAM in the same whole minute (n = 2838)	
**Patient demographics**								
Age, median (IQR), y	69 (58-79)	70 (59-80)	71 (58-80)	0.045	79 (69-86)	80 (70-87)	80 (71-87)	0.054
Sex								
Male	4963 (79.8)	11 464 (76.5)	313 (80.1)	0.057	24 340 (58.7)	109 548 (56.7)	1661 (58.5)	0.028
Female	1258 (20.2)	3516 (23.5)	78 (19.9)		17 095 (41.3)	83 824 (43.3)	1177 (41.5)	
**Cardiac arrest characteristics**								
Year								
2014	950 (15.3)	2486 (16.6)	56 (14.3)	0.111	4956 (12.0)	31 648 (16.4)	355 (12.5)	0.153
2015	996 (16.0)	2313 (15.4)	61 (15.6)		5422 (13.1)	30 984 (16.0)	357 (12.6)	
2016	1058 (17.0)	2438 (16.3)	62 (15.9)		6041 (14.6)	30 692 (15.9)	389 (13.7)	
2017	1079 (17.3)	2536 (16.9)	71 (18.2)		7000 (16.9)	32 385 (16.7)	478 (16.8)	
2018	1053 (16.9)	2635 (17.6)	55 (14.1)		8343 (20.1)	34 135 (17.7)	574 (20.2)	
2019	1085 (17.4)	2572 (17.2)	86 (22.0)		9673 (23.3)	33 528 (17.3)	685 (24.1)	
Season								
Spring	1471 (23.6)	3585 (23.9)	95 (24.3)	0.053	9924 (24.0)	47 261 (24.4)	701 (24.7)	0.035
Summer	1384 (22.2)	3333 (22.2)	93 (23.8)		8284 (20.0)	36 014 (18.6)	539 (19.0)	
Fall	1461 (23.5)	3575 (23.9)	97 (24.8)		9625 (23.2)	43 573 (22.5)	624 (22.0)	
Winter	1905 (30.6)	4487 (30.0)	106 (27.1)		13 602 (32.8)	66 524 (34.4)	974 (34.3)	
Day								
Weekday (Monday-Friday)	4363 (70.1)	10 595 (70.7)	276 (70.6)	0.009	29 406 (71.0)	136 479 (70.6)	2043 (72.0)	0.021
Weekend (Saturday and Sunday)	1858 (29.9)	4385 (29.3)	115 (29.4)		12 029 (29.0)	56 893 (29.4)	795 (28.0)	
Time								
Daytime (9:00 am to 4:59 pm)	2674 (43.0)	6227 (41.6)	172 (44.0)	0.033	15 792 (38.1)	67 183 (34.7)	1103 (38.9)	0.057
Nighttime (5:00 pm to 8:59 am)	3547 (57.0)	8753 (58.4)	219 (56.0)		25 643 (61.9)	126 189 (65.3)	1735 (61.1)	
Etiology								
Medical	6088 (97.9)	14 637 (97.7)	383 (98.0)	0.011	37 333 (90.1)	173 833 (89.9)	2578 (90.8)	0.021
Nonmedical	133 (2.1)	343 (2.3)	8 (2.0)		4102 (9.9)	19 539 (10.1)	260 (9.2)	
Witness status								
Unwitnessed	1827 (29.4)	4946 (33.0)	112 (28.6)	0.063	19 722 (47.6)	124 840 (64.6)	1453 (51.2)	0.231
Witnessed	4394 (70.6)	10 034 (67.0)	279 (71.4)		21 713 (52.4)	68 532 (35.4)	1385 (48.8)	
Initial rhythm								
PEA	NA	NA	NA	NA	15 736 (38.0)	46 042 (23.8)	1009 (35.6)	0.207
Asystole	NA	NA	NA		25 699 (62.0)	147 330 (76.2)	1829 (64.4)	
**Bystander interventions**								
CPR								
Chest compression only	3360 (54.0)	7850 (52.4)	221 (56.5)	0.093	19 870 (48.0)	92 310 (47.7)	1449 (51.1)	0.052
Chest compression with ventilation	488 (7.8)	1357 (9.1)	40 (10.2)		2497 (6.0)	11 068 (5.7)	175 (6.2)	
None	2373 (38.1)	5773 (38.5)	130 (33.2)		19 068 (46.0)	89 994 (46.5)	1214 (42.8)	
Public access AED shock delivery	869 (14.0)	2424 (16.2)	64 (16.4)	0.045	NA	NA	NA	NA
**EMS interventions**								
Dispatcher CPR instruction	3680 (59.2)	8988 (60.0)	256 (65.5)	0.087	25 128 (60.6)	124 092 (64.2)	1865 (65.7)	0.070
Prehospital physician involvement	395 (6.3)	823 (5.5)	16 (4.1)	0.068	1871 (4.5)	4530 (2.3)	99 (3.5)	0.080
Interval between emergency call and initiation of EMS CPR, median (IQR), min	9 (7-11)	9 (7-11)	9 (7-11)	0.098	9 (7-11)	9 (7-11)	9 (7-11)	0.093
Epinephrine administration	6221 (100.0)	7088 (47.3)	391 (100.0)	0.995	41 435 (100.0)	68 074 (35.2)	2838 (100.0)	1.279
Interval between initiation of EMS CPR and epinephrine administration, median (IQR), min	11 (8-15)	14 (10-19)	8 (6-12)	0.648	12 (9-16)	15 (10-20)	9 (6-12)	0.691
Type of AAM								
SGA	1513 (24.3)	13 071 (87.3)	301 (77.0)	1.512	8782 (21.2)	157 858 (81.6)	1929 (68.0)	1.580
ETI	399 (6.4)	1909 (12.7)	90 (23.0)		3330 (8.0)	35 514 (18.4)	909 (32.0)	
No AAM	4309 (69.3)	0 (0.0)	0 (0.0)		29 323 (70.8)	0 (0.0)	0 (0.0)	
Interval between initiation of EMS CPR and AAM, median (IQR), min	14 (10-19)	7 (5-11)	8 (6-12)	0.839	14 (10-19)	8 (5-11)	9 (6-12)	0.761

Abbreviations: AAM, advanced airway management; AED, automated external defibrillator; CPR, cardiopulmonary resuscitation; EMS, emergency medical services; ETI, endotracheal intubation; NA, not applicable; PEA, pulseless electrical activity; SGA, supraglottic airway.

^a^ Standardized mean difference of variables between the epinephrine-first and AAM-first groups.

Using IPTW, we successfully balanced all covariates between the epinephrine-first group and AAM-first group, with all standardized mean differences in the weighted shockable and nonshockable subcohorts less than 0.100 (Table 2). After IPTW, in the shockable rhythm subcohort, the median (IQR) time from the initiation of CPR by EMS personnel to epinephrine administration was 9 (6-13) minutes in the epinephrine-first group and 15 (11-20) minutes in the AAM-first group, and the median (IQR) time from the initiation of CPR by EMS personnel to AAM was 12 (8-16) minutes in the epinephrine-first group and 8 (5-13) minutes in the AAM-first group. In the nonshockable rhythm subcohort, the median (IQR) time to epinephrine administration was 9 (6-13) minutes in the epinephrine-first group and 15 (11-21) minutes in the AAM-first group, and the median (IQR) time to AAM was 12 (9-16) minutes in the epinephrine-first group and 8 (5-12) minutes in the AAM-first group.

**Table 2.** Characteristics of Adult Patients With Out-of-Hospital Cardiac Arrest After Inverse Probability of Treatment Weighting^a^

	Patients, %					
	Shockable			Nonshockable		
	Epinephrine first (n = 20 167)	AAM first (n = 21 888)	Standardized mean difference^b^	Epinephrine first (n = 217 018)	AAM first (n = 238 102)	Standardized mean difference^b^
**Patient demographics**						
Age, median (IQR), y	69 (58-80)	70 (58-80)	0.001	80 (70-87)	80 (70-87)	0.006
Sex						
Male	77.5	77.7	0.004	57.2	57.0	0.004
Female	22.5	22.3		42.8	43.0	
**Cardiac arrest characteristics**						
Year						
2014	15.9	15.9	0.010	14.5	15.5	0.041
2015	15.3	15.6		14.6	15.4	
2016	16.6	16.6		15.5	15.6	
2017	17.2	17.3		17.1	16.8	
2018	17.7	17.7		18.9	18.2	
2019	17.3	17.0		19.3	18.6	
Season						
Spring	23.8	24.0	0.013	24.3	24.3	0.001
Summer	22.4	22.0		18.9	18.9	
Fall	23.6	23.9		22.6	22.6	
Winter	30.2	30.1		34.2	34.1	
Day						
Weekday (Monday-Friday)	70.2	70.8	0.012	70.7	70.6	0.001
Weekend (Saturday and Sunday)	29.8	29.2		29.3	29.4	
Time						
Daytime (9:00 am to 4:59 pm)	41.8	41.5	0.007	35.5	35.4	0.003
Nighttime (5:00 pm to 8:59 am)	58.2	58.5		64.5	64.6	
Etiology						
Medical	97.4	97.8	0.024	89.4	89.8	0.014
Nonmedical	2.6	2.2		10.6	10.2	
Witness status						
Unwitnessed	31.9	31.8	0.002	59.5	61.2	0.036
Witnessed	68.1	68.2		40.5	38.8	
Initial rhythm						
PEA	NA	NA	NA	27.7	26.5	0.026
Asystole	NA	NA		72.3	73.5	
**Bystander interventions**						
CPR			0.002			0.015
Chest compression only	53.0	53.1		48.5	47.8	
Chest compression with ventilation	8.7	8.7		5.7	5.8	
None	38.3	38.2		45.8	46.5	
Public access AED shock delivery	15.8	15.9	0.003	NA	NA	NA
**EMS interventions**						
Dispatcher CPR instruction	60.1	59.7	0.007	63.4	63.5	0.001
Prehospital physician involvement	5.8	5.9	0.005	2.9	2.8	0.008
Interval between emergency call and initiation of EMS CPR, median (IQR), min	9 (7-11)	9 (7-11)	0.001	9 (7-11)	9 (7-11)	0.019
Interval between EMS CPR and first treatment (epinephrine or AAM), median (IQR), min	9 (6-13)	8 (5-13)	0.023	9 (6-13)	8 (5-12)	0.090

Abbreviations: AAM, advanced airway management; AED, automated external defibrillator; CPR, cardiopulmonary resuscitation; EMS, emergency medical services; NA, not applicable; PEA, pulseless electrical activity.

^a^ Excluding patients who received epinephrine and AAM in the same whole minute.

^b^ Standardized mean difference of variables between the epinephrine-first and AAM-first groups.

In the IPTW analysis, patients receiving epinephrine first had an increased likelihood of 1-month survival among those with an initial shockable rhythm (odds ratio [OR], 1.19; 95% CI, 1.09-1.30) and with an initial nonshockable rhythm (OR, 1.28; 95% CI, 1.19-1.37) compared with patients receiving AAM first (Table 3). Patients receiving epinephrine first also had an increased likelihood of 1-month survival with favorable functional status among those with an initial shockable rhythm (OR, 1.24; 95% CI, 1.10-1.39) and with an initial nonshockable rhythm (OR, 1.39; 95% CI, 1.17-1.64). The epinephrine-first group had an increased likelihood of prehospital ROSC after an initial shockable rhythm (OR, 1.74; 95% CI, 1.61-1.88) and nonshockable rhythm (OR, 2.59; 95% CI, 2.50-2.68).

**Table 3.** Results of Inverse Probability of Treatment Weighting Analyses

Outcome	Patients with outcome, No./total No. (%)		OR (95% CI)^a^
	Epinephrine first	AAM first	
**Primary analysis** ^b^			
Shockable rhythm			
1-mo Survival	3924/20 167 (19.5)	3692/21 888 (16.9)	1.19 (1.09-1.30)
1-mo Survival with favorable functional status	1969/20 167 (9.8)	1763/21 888 (8.1)	1.24 (1.10-1.39)
Prehospital ROSC	5475/20 167 (27.1)	3858/21 888 (17.6)	1.74 (1.61-1.88)
Nonshockable rhythm			
1-mo Survival	6449/217 018 (3.0)	5578/238 102 (2.3)	1.28 (1.19-1.37)
1-mo Survival with favorable functional status	1160/217 018 (0.5)	919/238 102 (0.4)	1.39 (1.17-1.64)
Prehospital ROSC	39 652/217 018 (18.3)	18 950/238 102 (8.0)	2.59 (2.50-2.68)
**Sensitivity analysis 1** ^c^			
Shockable rhythm			
1-mo Survival	4002/20 551 (19.5)	3785/22 239 (17.0)	1.18 (1.08-1.28)
1-mo Survival with favorable functional status	2009/20 551 (9.8)	1797/22 239 (8.1)	1.23 (1.10-1.38)
Prehospital ROSC	5582/20 551 (27.2)	3985/22 239 (17.9)	1.71 (1.58-1.85)
Nonshockable rhythm			
1-mo Survival	6549/219 780 (3.0)	5764/240 835 (2.4)	1.25 (1.17-1.35)
1-mo Survival with favorable functional status	1179/219 780 (0.5)	948/240 835 (0.4)	1.36 (1.15-1.62)
Prehospital ROSC	40 214/219 780 (18.3)	19 838/240 835 (8.2)	2.49 (2.41-2.58)
**Sensitivity analysis 2** ^d^			
Shockable rhythm			
1-mo Survival	4047/20 667 (19.6)	3761/22 236 (16.9)	1.20 (1.10-1.30)
1-mo Survival with favorable functional status	1989/20 667 (9.6)	1796/22 236 (8.1)	1.21 (1.08-1.35)
Prehospital ROSC	5624/20 667 (27.2)	3927/22 236 (17.7)	1.74 (1.62-1.88)
Nonshockable rhythm			
1-mo Survival	6982/221 992 (3.1)	5660/240 696 (2.4)	1.35 (1.26-1.44)
1-mo Survival with favorable functional status	1240/221 992 (0.6)	933/240 696 (0.4)	1.44 (1.23-1.70)
Prehospital ROSC	41 778/221 992 (18.8)	19 220/240 696 (8.0)	2.67 (2.58-2.76)

Abbreviations: AAM, advanced airway management; OR, odds ratio; ROSC, return of spontaneous circulation.

^a^ The reference group was patients who received AAM first.

^b^ Excluding patients who received epinephrine and AAM within the same whole minute.

^c^ Patients who received epinephrine and AAM within the same whole minute were included in AAM-first group.

^d^ Patients who received epinephrine and AAM within the same whole minute were included in epinephrine-first group.

In the 2 sensitivity analyses, IPTW using propensity scores balanced all covariates between the epinephrine-first group and AAM-first group (eTables 1 and 2 in Supplement 1), with all standardized mean differences in the weighted shockable and nonshockable subcohorts of less than 0.100. Across the sensitivity analyses, the findings were similar to those in the primary analysis, showing for both initial shockable and nonshockable rhythms, respectively, associations of epinephrine first with 1-month survival (OR, 1.20 [95% CI, 1.10-1.30] and 1.35 [95% CI, 1.26-1.44]), 1-month survival with favorable functional status (OR, 1.21 [95% CI, 1.08-1.35] and 1.44 [95% CI, 1.23-1.70]), and prehospital ROSC (OR, 1.74 [95% CI, 1.62-1.88] and 2.67 [95% CI, 2.58-2.76]) (Table 3).

## Discussion

In our analyses of the nationwide, population-based OHCA registry comprising 259 237 adult patients in Japan who received epinephrine and/or AAM, we found that the epinephrine-first strategy was associated with an increased likelihood of 1-month survival, 1-month survival with favorable functional status, and prehospital ROSC for both shockable and nonshockable rhythms compared with the AAM-first strategy. These findings remained consistent in sensitivity analyses.

The existing literature has not established a definitive relationship between the timing of epinephrine administration and survival or functional outcomes after OHCA. A 2019 systematic review examining vasopressors during adult cardiac arrest identified 16 observational studies of the timing of epinephrine administration.^25^ However, the review reported that differences in survival to hospital discharge and favorable neurologic outcomes were constrained by very low event rates and inconsistent results across studies. More recently, evidence of the timing of epinephrine has been accumulated and has supported early epinephrine administration. For example, a secondary analysis in 2020 of a clinical trial comparing epinephrine with placebo, which included more than 4800 adult OHCAs, showed a reduction in the rates of ROSC, survival, and favorable functional outcomes associated with longer durations of cardiac arrest and that earlier administration of epinephrine was associated with better outcomes.^26^ Similarly, a retrospective analysis in 2021 of Resuscitation Outcomes Consortium (ROC) findings showed that among more than 40 000 adult patients with OHCA, survival to hospital discharge and favorable functional outcome varied with the timing of epinephrine administration and decreased with delayed epinephrine administration for shockable and nonshockable rhythms.^27^

Conversely, evidence surrounding the timing of AAM remains more inconclusive. An observational study of a population-based OHCA registry in Osaka, Japan, showed that increased time from the initiation of CPR by EMS personnel to AAM was associated with a lower likelihood of 1-month survival and favorable functional status at 1 month.^28^ An observational study of the ROC Prehospital Resuscitation Using an Impedance Valve and Early vs Delayed trial reported that early AAM was associated with prehospital ROSC.^29^ In contrast, a retrospective analysis of the Pragmatic Airway Resuscitation Trial, which compared laryngeal tube and endotracheal intubation, demonstrated that the timing of the AAM attempt was not associated with survival to hospital discharge.^30^ Given the available evidence, the 2021 European Resuscitation Council guidelines support a stepwise approach of airway management for cardiac arrest, which implies that multiple devices (ie, basic and advanced airway devices) may be used during a single resuscitation attempt.^31^ However, to our knowledge, none of the previous studies directly compared the sequence of epinephrine administration and AAM.

Our findings support early epinephrine administration prior to AAM for adults with OHCA and suggest that the benefits of epinephrine may be more time dependent than those of AAM. A clinical trial comparing IV or IO epinephrine administration with saline placebo for OHCA demonstrated a favorable survival outcome with epinephrine (OR for 30-day survival, 1.39; 95% CI, 1.06-1.82).^32^ In contrast, prior studies did not show the superiority of AAM for survival or favorable functional outcomes compared with bag-valve-mask ventilation.^33,34^ Our study results, along with the currently available evidence, suggest that epinephrine administration may be more beneficial than AAM for adult patients with OHCA, indicating that prioritizing epinephrine administration over AAM might be reasonable.

Nevertheless, our study results should be interpreted as the estimated magnitude of the association between the sequence of epinephrine and placement of an advanced airway and outcomes within the study population and EMS setting, which comprise different characteristics from other health care settings. In our study population, only 28.8% of patients with shockable rhythm and 17.4% of those with nonshockable rhythm received epinephrine first, illustrating that the majority of patients underwent AAM first. In an observational study of the ROC, a multicenter clinical research network in the US and Canada, the median (IQR) intervals between EMS arrival and the first epinephrine administration were 7.3 (5.3-10.0) minutes for shockable rhythm and 8.1 (6.0-11.0) minutes for nonshockable rhythm, whereas the time to AAM was 10 minutes for both shockable and nonshockable rhythms.^27^ This observation suggests that for the majority of patients in the ROC, epinephrine was administered earlier than AAM. This discrepancy may imply differences in prehospital care practice between Japan and North America, potentially explained by variations in vascular access across the study settings. In Japan, the IV route was the sole vascular access, while in the ROC, the IO route served as an alternative vascular access. In another analysis of the ROC dataset, 27 758 of 35 733 (77.7%) adult patients received IV epinephrine, and 7975 (22.3%) received IO epinephrine.^35^ A clinical trial comparing the success rates of vascular access between IO and IV routes of administration showed that tibial IO had a greater initial success rate (95% [95% CI, 90%-100%] vs 49% [95% CI, 37%-61%]) and a shorter interval between EMS arrival and successful vascular access (4.6 [IQR, 3.6-6.2] minutes vs 5.8 [IQR, 4.1-8.0] minutes).^36^ Since the IV route was the only vascular access in the current study, it might not have been feasible to establish IV access and administer epinephrine before AAM. Further work may be warranted to assess the sequence of epinephrine administration and AAM in other health care settings that use a different epinephrine administration strategy.

### Limitations

Our study has several limitations. First, the sequence of epinephrine administration and AAM was not randomly determined but at the discretion of EMS personnel under online medical direction, potentially introducing confounding by indication.^37^ The time required for successful vascular access and advanced airway placement may have been the primary determinant of the sequence of interventions. Consequently, the study results should not be interpreted as establishing a causal relationship. Second, the registry did not capture unsuccessful IV or advanced airway placement attempts. As a result, it is unclear what proportions of patients in the epinephrine-first group experienced failed AAM before epinephrine administration and how many patients in the AAM-first group experienced failed IV access before advanced airway placement. It is possible that some patients received one intervention as a consequence of a failed attempt of the other intervention. Similarly, data were not available for specific patient characteristics (eg, obesity) and numbers of ELSTs, which may have influenced the sequence and timing of epinephrine administration and AAM. Third, postresuscitation treatments (eg, targeted temperature management, hemodynamic and ventilator management, coronary revascularization, prognostication) were not available in the dataset. Differences in strategies for postresuscitation treatment may have influenced survival and functional outcomes.

## Conclusions

The findings of this cohort study suggest that administration of IV epinephrine first for adult OHCA is associated with an increased likelihood of 1-month survival, 1-month survival with favorable functional status, and prehospital ROSC among Japanese patients with shockable and nonshockable rhythms compared with an AAM-first strategy. Thus, this study supports epinephrine administration before placement of an advanced airway as the optimal sequence of intervention for OHCA.

## Notes

Supplement 1. **eTable 1.** Characteristics of Adult Patients With Out-of-Hospital Cardiac Arrest After Inverse Probability of Treatment Weighting (Sensitivity Analysis 1) **eTable 2.** Characteristics of Adult Patients With Out-of-Hospital Cardiac Arrest After Inverse Probability of Treatment Weighting (Sensitivity Analysis 2)

Supplement 2.
Data Sharing Statement
