# Supplementary material for: Sequence of Epinephrine and Advanced Airway Placement After Out-of-Hospital Cardiac Arrest
Source: JAMA Netw Open. 2024 Feb 19;7(2):e2356863. doi: 10.1001/jamanetworkopen.2023.56863 (PMC10877448; doi:10.1001/jamanetworkopen.2023.56863)
Supplement: Supplement 1. — eTable 1. Characteristics of Adult Patients With Out-of-Hospital Cardiac Arrest After Inverse Probability of Treatment Weighting (Sensitivity Analysis 1) eTable 2. Characteristics of Adult Patients With Out-of-Hospital Cardiac Arrest After Inverse Probability of Treatment Weighting (Sensitivity Analysis 2) [file jamanetwopen-e2356863-s001.pdf]

Supplemental Online Content

Okubo M, Komukai S, Izawa J, et al. Sequence of epinephrine and placement of an advanced airway and outcome after out-of-hospital cardiac arrest. *JAMA Netw Open*. 2024;7(2):e2356863. doi:10.1001/jamanetworkopen.2023.56863

eTable 1. Characteristics of Adult Patients With Out-of-Hospital Cardiac Arrest After Inverse Probability of Treatment Weighting (Sensitivity Analysis 1)

eTable 2. Characteristics of Adult Patients With Out-of-Hospital Cardiac Arrest After Inverse Probability of Treatment Weighting (Sensitivity Analysis 2)

This supplemental material has been provided by the authors to give readers additional information about their work.

© 2024 Okubo M et al. *JAMA Network Open.*

eTable 1. Characteristics of Adult Patients With Out-of-Hospital Cardiac Arrest After Inverse Probability of Treatment Weighting (Sensitivity Analysis 1)[a]

	Shockable			Nonshockable		
	Epinephrine 1st (n=20551)	AAM 1st (n=22239)	Standardized mean difference	Epinephrine 1st (n=219780)	AAM 1st (n=240835)	Standardized mean difference
**Patient demographics**						
Age, median (IQR), years	69 (58-80)	70 (58-80)	0.002	80 (70-87)	80 (70-87)	0.006
Sex, %			0.003			0.004
Male	77.6	77.7		57.3	57.1	
Female	22.4	22.3		42.7	42.9	
**Arrest characteristics**						
Year of arrest, %			0.011			0.041
2014	15.9	15.8		14.5	15.5	
2015	15.3	15.6		14.6	15.4	
2016	16.6	16.6		15.5	15.5	
2017	17.2	17.3		17.1	16.8	
2018	17.6	17.6		18.9	18.2	
2019	17.4	17.1		19.4	18.6	
Season of arrest, %			0.013			0.001
Spring	23.8	24.0		24.3	24.3	
Summer	22.4	22.0		18.9	18.9	
Fall	23.6	24.0		22.6	22.6	
Winter	30.1	30.0		34.2	34.1	
Day of arrest, %			0.012			0.001
Weekday (Monday to Friday)	70.2	70.7		70.7	70.6	

© 2024 Okubo M et al. *JAMA Network Open.*

Weekend (Saturday and Sunday)	29.8	29.3		29.3	29.4	
Time of arrest, %			0.006			0.003
Daytime (9:00 to 16:59)	41.9	41.6		35.6	35.4	
Nighttime (17:00 to 8:59)	58.1	58.4		64.4	64.6	
Etiology, %			0.022			0.014
Medical	97.4	97.8		89.4	89.9	
Non-medical	2.6	2.2		10.6	10.1	
Witness status, %			0.002			0.036
Unwitnessed	31.8	31.8		59.4	61.1	
Witnessed	68.2	68.2		40.6	38.9	
Initial rhythms, %			N/A			0.026
PEA	N/A	N/A		27.8	26.6	
Asystole	N/A	N/A		72.2	73.4	
**Bystander interventions**						
Bystander CPR, %			0.001			0.015
Chest compression only CPR	53.0	53.1		48.6	47.8	
Chest compression with ventilation	8.7	8.7		5.7	5.8	
No bystander CPR	38.2	38.2		45.8	46.4	
Public access AED shock delivery, %	15.8	15.9	0.002	N/A	N/A	N/A
**EMS interventions**						
Dispatcher CPR instruction, %	60.1	59.8	0.007	63.5	63.5	0.001
Prehospital physician involvement, %	5.8	5.9	0.005	2.9	2.8	0.009
Interval between emergency call and initiation of EMS CPR, median (IQR), minutes	9 (7-11)	9 (7-11)	0.002	9 (7-11)	9 (7-11)	0.019

© 2024 Okubo M et al. *JAMA Network Open.*

Interval between EMS CPR and 1st treatment (epinephrine or AAM), median (IQR), minutes	9 (6-12.5)	8 (5-13)	0.015	9 (6-13)	8 (5-12)	0.093

[a] Patients who received epinephrine and advanced airway management within the same whole minutes were included in advanced airway management 1st group.

Abbreviations: AAM, advanced airway management; AED, automated external defibrillator; CPR, cardiopulmonary resuscitation; EMS, emergency medical services; IQR, interquartile range; N/A, not applicable; PEA, pulseless electrical activity.

© 2024 Okubo M et al. *JAMA Network Open.*

eTable 2. Characteristics of Adult Patients With Out-of-Hospital Cardiac Arrest After Inverse Probability of Treatment Weighting (Sensitivity Analysis 2)[a]

	Shockable			Nonshockable		
	Epinephrine 1st (n=20667)	AAM 1st (n=22236)	Standardized mean difference	Epinephrine 1st (n=221992)	AAM 1st (n=240696)	Standardized mean difference
**Patient demographics**						
Age, median (IQR), years	70 (58-80)	70 (58-80)	0.002	80 (70-87)	80 (70-87)	0.004
Sex, %			0.003			0.003
Male	77.6	77.7		57.2	57.1	
Female	22.4	22.3		42.8	42.9	
**Arrest characteristics**						
Year of arrest, %			0.011			0.037
2014	15.9	15.9		14.6	15.5	
2015	15.3	15.6		14.7	15.4	
2016	16.6	16.6		15.4	15.5	
2017	17.2	17.3		17.1	16.8	
2018	17.5	17.5		18.9	18.2	
2019	17.5	17.1		19.2	18.6	
Season of arrest, %			0.013			0.001
Spring	23.7	24.0		24.3	24.3	
Summer	22.5	22.0		18.9	18.9	
Fall	23.7	24.0		22.6	22.6	
Winter	30.1	30.0		34.2	34.1	
Day of arrest, %			0.011			0.001
Weekday (Monday to Friday)	70.2	70.7		70.7	70.6	
Weekend (Saturday and Sunday)	29.8	29.3		29.3	29.4	

© 2024 Okubo M et al. *JAMA Network Open.*

Time of arrest, %			0.008			0.002
Daytime (9:00 to 16:59)	42.0	41.6		35.5	35.4	
Nighttime (17:00 to 8:59)	58.0	58.4		64.5	64.6	
Etiology, %			0.020			0.013
Medical	97.5	97.8		89.5	89.9	
Non-medical	2.5	2.2		10.5	10.1	
Witness status, %			0.001			0.032
Unwitnessed	31.8	31.8		59.5	61.1	
Witnessed	68.2	68.2		40.5	38.9	
Initial rhythms, %			N/A			0.024
PEA	N/A	N/A		27.7	26.6	
Asystole	N/A	N/A		72.3	73.4	
**Bystander interventions**						
Bystander CPR, %			0.003			0.015
Chest compression only CPR	53.2	53.1		48.6	47.8	
Chest compression with ventilation	8.7	8.8		5.7	5.8	
No bystander CPR	38.1	38.1		45.7	46.4	
Public access AED shock delivery, %	15.8	15.9	0.003	N/A	N/A	N/A
**EMS interventions**						
Dispatcher CPR instruction, %	60.3	59.9	0.009	63.5	63.5	<0.001
Prehospital physician involvement, %	5.7	5.8	0.006	2.9	2.8	0.008
Interval between emergency call and initiation of EMS CPR, median (IQR), minutes	9 (7-11)	9 (7-11)	0.003	9 (7-11)	9 (7-11)	0.014

© 2024 Okubo M et al. *JAMA Network Open.*

Interval between EMS CPR and 1st treatment (epinephrine or AAM), median (IQR), minutes	9 (6-12)	8 (5-13)	0.024	9 (6-13)	8 (5-12)	0.079

[a] Patients who received epinephrine and advanced airway management within the same whole minutes were included in epinephrine 1st group.

Abbreviations: AAM, advanced airway management; AED, automated external defibrillator; CPR, cardiopulmonary resuscitation; EMS, emergency medical services; IQR, interquartile range; N/A, not applicable; PEA, pulseless electrical activity.

© 2024 Okubo M et al. *JAMA Network Open.*
