# Supplementary material for: Sequence of Epinephrine and Advanced Airway Placement After Out-of-Hospital Cardiac Arrest
Source: JAMA Netw Open. 2024 Feb 19;7(2):e2356863. doi: 10.1001/jamanetworkopen.2023.56863 (PMC10877448; doi:10.1001/jamanetworkopen.2023.56863)
Supplement: Supplement 2. — Data Sharing Statement [file jamanetwopen-e2356863-s002.pdf]

Data Sharing Statement

Okubo. Sequence of Epinephrine and Advanced Airway Placement After Out-of-Hospital Cardiac Arrest. *JAMA Netw Open*. Published February 19, 2024.
doi:10.1001/jamanetworkopen.2023.56863

Data
Data available: No
